# Nrf2/ARE Signaling Pathway: Key Mediator in Oxidative Stress and Potential Therapeutic Target in ALS

**DOI:** 10.1155/2012/878030

**Published:** 2012-09-20

**Authors:** Susanne Petri, Sonja Körner, Mahmoud Kiaei

**Affiliations:** ^1^Department of Neurology, Hannover Medical School, Hannover, Germany; ^2^Department of Neurobiology and Developmental Sciences, Center for Translational Neuroscience, University of Arkansas for Medical Sciences, Little Rock, AR, USA

## Abstract

Nrf2 (nuclear erythroid 2-related factor 2) is a basic region leucine-zipper transcription factor which binds to the antioxidant response element (ARE) and thereby regulates the expression of a large battery of genes involved in the cellular antioxidant and anti-inflammatory defence as well as mitochondrial protection. As oxidative stress, inflammation and mitochondrial dysfunctions have been identified as important pathomechanisms in amyotrophic lateral sclerosis (ALS), this signaling cascade has gained interest both with respect to ALS pathogenesis and therapy. Nrf2 and Keap1 expressions are reduced in motor neurons in postmortem ALS tissue. Nrf2-activating compounds have shown therapeutic efficacy in the ALS mouse model and other neurodegenerative disease models. Alterations in Nrf2 and Keap1 expression and dysregulation of the Nrf2/ARE signalling program could contribute to the chronic motor neuron degeneration in ALS and other neurodegenerative diseases. Therefore, Nrf2 emerges as a key neuroprotective molecule in neurodegenerative diseases. Our recent studies strongly support that the Nrf2/ARE signalling pathway is an important mediator of neuroprotection and therefore represents a promising target for development of novel therapies against ALS, Parkinson's disease (PD), Huntington's disease (HD), and Alzheimer's disease (AD).

## 1. Introduction

Amyotrophic lateral sclerosis (ALS) is the most common adult-onset motor neuron disease. It causes degeneration of motor neurons in the primary motor cortex, brain stem, and spinal cord which subsequently leads to rapidly progressive paralysis of skeletal muscles and ultimately to death due to respiratory failure, usually within 3 to 5 years after disease onset.

The majority of ALS cases are acquired spontaneously (sporadic ALS; sALS), while only 10%–15% of ALS cases are inherited (familial ALS; fALS) [1]. Recent breakthroughs in genetics have enlarged the number of known mutations causing fALS, among them mutations in genes coding for superoxide dismutase 1 (SOD1), TAR DNA-binding protein (TARDP), fused-in sarcoma/translocation in liposarcoma (FUS/TLS), and, most recently, a repeat expansion of C9orf72, the cause of chromosome 9-linked ALS and frontotemporal lobar dementia (FTLD) [2]. Mutation in the gene for profilin1 (PFN1) was recently reported to be associated with several fALS families. Four mutations (C71G, M114T, E117G, and G118V) were identified in several families [3]. The etiology of sALS is less clear and must be considered multifactorial and polygenic in the majority of cases. 

Several interdependent and interacting mechanisms have been shown to induce motor neuron damage in both fALS and sALS: excitotoxicity, aberrant RNA processing, altered axonal transport, protein aggregation, mitochondrial dysfunction, toxicity of nonneuronal (glial) cells and oxidative stress [4]. Even if it is unclear whether oxidative stress is a primary or a secondary cause of neurodegeneration in ALS, data from both human tissue and studies in transgenic animal models suggest that it is a major contributory factor leading to chronic motor neuron death. In mutant superoxide dismutase 1 (SOD1) ALS-mouse models [5] as well as in human familial and sporadic ALS, markers of oxidative damage of proteins, lipids and DNA are elevated in brain and spinal cord [6–9]. A larger number of nonspecific antioxidants (e.g., creatine, coenzyme Q 10, vitamine E, N-acetyl-cysteine, and others) have been tested in transgenic mouse models for ALS and were efficient regarding survival, disease progression and motor neuron loss in the spinal cord. Clinical trials in ALS patients have not yet been able to prove efficacy of antioxidant treatment in the clinical setting, but ongoing trials such as the dexpramipexole study still use compounds with antioxidant potential which underlines the importance and acceptance of this therapeutic strategy [10].

**Figure 1:**
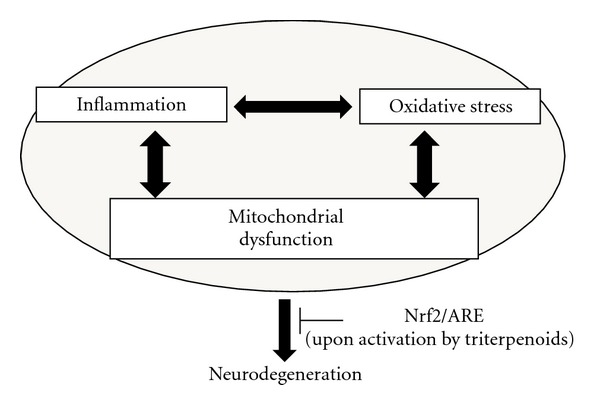
Three major toxic pathways that contribute to neurodegeneration. Nrf2/ARE signaling activation via triterpenoids can reduce oxidative damage, lessen inflammation, and restore mitochondria resulting in more robust motor neurons able to defend themselves against toxic insults.

Inflammation and mitochondrial dysfunction are considered as major pathomechanisms in motor neuron degeneration. These processes both have strong interconnections to oxidative stress cascades. These three pathways are overlapping and interconnected and appear to form a vicious cycle that each could be an initiator as well as a mediator of motor neuron death (Figure 1). 

Mitochondrial injury is known to result in an excess of oxygen radicals. Depending on the cell type whose mitochondria are injured, that may trigger different sets of reactions. For instance, glial cells with injured mitochondria can produce proinflammatory molecules that could be toxic to neurons and other cells nearby. These types of inflammatory reactions are stressors for neurons and will be adding to oxidative stress that neurons are subject to. All these in turn will be damaging to mitochondria and other cellular organelles which may lead to further inflammatory reactions in neurons and other cells in CNS (see review by Sun et al. [11]).

A key molecule regulating the cellular antioxidant response is the basic region leucine-zipper transcription factor Nrf2 (nuclear factor erythroid 2-related factor 2). In basal conditions, Nrf2 is bound to the endogenous inhibitor Kelch-like ECH associated protein1 (Keap1). Once activated, it translocates to the nucleus of the cell where it forms heterodimers with other transcription factors such as c-Jun and small Maf proteins (G/F/K), binds to the antioxidant response element (ARE), a regulatory enhancer region within gene promoters. c-Jun is then supposed to act mainly as transcriptional activator while the small Mafs as well as c-Myc inactivate gene transcription after Nrf2 binding [12]. Nrf2-ARE binding regulates the expression of more than 200 genes involved in the cellular antioxidant and anti-inflammatory defense such as phase 2 detoxification enzymes (NAD(P)H quinone oxyreductase, glutathione), enzymes which are necessary for glutathione biosynthesis, extracellular superoxide dismutase, glutamate-6-phosphate-dehydrogenase, heat shock proteins and ferritin, furthermore pro- und anti-inflammatory enzymes such as cyclooxygenase-2 (COX-2), inducible nitric oxide synthase (iNOS), and heme oxygenase-1 (HO-1) [13–15]. Nrf2 has also been reported to regulate the expression of genes promoting mitochondrial biogenesis such as mitochondrial transcription factors (TFAM) and is therefore directly involved in mitochondrial preservation [16]. 

Induction of Nrf2 by compounds of different chemical classes was shown to be directly correlated to the inhibition of proinflammatory responses (Cox-2 and iNOS expression), but the anti-inflammatory effects of these molecules are only partially Nrf2-dependent and the exact relation between Nrf2-induction and anti-inflammatory properties remains to be clarified [17, 18]. Probably by regulation of intracellular glutathione content, Nrf2 further has direct cytoprotective effects via the inhibition of Fas-mediated apoptotic pathways [15]. 

The endogenous inhibitor of Nrf2 is the actin-bound cytoskeletal zinc metalloprotein Keap1. Several models of interaction of Keap1 and Nrf2 have been proposed [18]. Modifications of cysteine residues of Keap1 apparently alter the interaction of Keap1 with Nrf2 and lead to its relocation to the cytoplasm where it is subsequently degraded by the ubiquitin-proteasome system [18–22]. Decreased Keap-Nrf2-binding (via oxidation of sulfhydryl groups or phosphorylation) results in intranuclear shuttling of Nrf2 and subsequent transcription of ARE-driven genes [13, 23–25]. Keap1 and Nrf2 therefore constitute a cellular sensor for damage caused by free oxygen radicals [21]. Some studies reported constant shuttling of Keap1 between the nucleus and the cytoplasm under physiological conditions. Karyopherin-6 (KPNA6) has been identified as a protein which facilitates nuclear import and attenuates Nrf2 signaling [26]. Furthermore, KPNA6 accelerates the clearance of Nrf2 protein from the nucleus, and even promotes the restoration of the Nrf2 protein to basal levels. These findings suggest that KPNA6-mediated Keap1 nuclear import plays an essential role in modulating the Nrf2-dependent antioxidant response and maintaining cellular redox homeostasis. 

In addition, it has also been shown that Nrf2 protein stability can be regulated in a Keap1-independent manner by phosphorylation via glycogen synthase kinase-3 (GSK-3*β*) [27] and that Nrf2 function can further be modified by regulation of its transcription [28].

There are multiple factors that activate Nrf2 in any given cell, for example, environmental stressors such as cigarette smoke, infection, oxidative stress, or inflammation. Several reports have shown that disruption of Nrf2 impairs the induction of the Nrf2/ARE pathway leading to exacerbation of oxidative stress, inflammation, and mitochondrial dysfunction (reviewed by [40]). Restorative effects of Nrf2 were reported in mice exposed to cigarette smoke [41].

## 2. Nrf2 in Other Neurodegenerative Diseases

Nrf2 protein expression has already been studied in postmortem brain tissue from patients with neurodegenerative diseases such as Alzheimer's (AD), Lewy body variant of AD (LBVAD), and Parkinson's disease (PD) [25]. Cytoplasmic localization of Nrf2 was found in AD and LBVAD hippocampi and entorhinal cortex. It was therefore concluded that in these diseases nuclear translocation of Nrf2 is impaired and that dysfunction in the Nrf2 pathway leads to decreased cellular defense against oxidative stress [25]. In the PD cases, in contrast, nuclear Nrf2 levels in the substantia nigra were increased which could be interpreted as an appropriate neuronal response to oxidative stimuli [25].

## 3. Nrf2-Cascade in ALS In Vitro Animal Models

Neuronal and astroglial primary cultures from Nrf2 knockout mice are more vulnerable to oxidative stress than wild-type cells, while overexpression of Nrf2 increases resistance against oxidative and excitotoxic stimuli [14, 42, 43]. 

Several studies have attempted to clarify the role of the Nrf2-pathway in SOD1-G93A transgenic ALS animal and in vitro models: reduced Nrf2-expression has been described in primary embryonic motor neuron cultures derived from SOD1-G93A transgenic mice. These SOD1-G93A-transgenic motor neurons were more sensitive to apoptosis induced by addition of nerve growth factor (NGF) [44]. In another in vitro model of ALS, motor neuron-like Nsc34 cells which were stably transfected with mutant SOD1, downregulation of genes regulated by Nrf2 was found by microarray analysis. The authors therefore suggested that pharmacological stimulation of the Nrf2-pathway could be a novel therapeutic approach in ALS [45]. Opposed to these in vitro studies in motor neurons, another group detected upregulated Nrf2-expression in astrocytes. This increased Nrf2-expression which was interpreted as reactive attempt to prevent cell death was already observed at disease onset and persisted throughout disease progression [46]. Activation of Nrf2-ARE signaling in ALS mice was studied by cross-breeding ALS-transgenic mice with ARE reporter mice. Thereby, ARE activation could be directly measured via induction of ARE driven hPAP (human placental alkaline phosphatase) activity. In this model, early and intense Nrf2-activation was seen in skeletal muscles. Comparably less activation of Nrf2 was seen in spinal cord motor neurons and astrocytes after symptom onset [47]. The authors therefore concluded that the earliest pathological events in mutant SOD1-associated ALS occur in muscle tissue and that they progress in a retrograde manner during the disease course.

## 4. Nrf2 in Human Sporadic ALS

While these mutant SOD1 models reproduce pathophysiology of familial ALS, no ideal model of sporadic ALS exists so far. We have recently investigated mRNA and protein expression of the transcription factor Nrf2 and its endogenous inhibitor Keap1 in postmortem brain and spinal cord specimens of sporadic ALS patients and controls. Reduced neuronal mRNA expression levels of Nrf2 were seen in motor neurons of the primary motor cortex and the ventral horn. Immunohistochemistry and Western blot experiments revealed correspondingly decreased Nrf2 protein expression. Astrocytosis was increased in ALS tissues. By colocalized immunohistochemistry, we observed some astrocytic localization of Nrf2 but an overall decreased Nrf2 protein expression as compared to control specimens. For the endogenous inhibitor Keap1, a slight but not significant increase in mRNA expression was observed in the motor cortex but not in the spinal cord. Keap1 protein expression was unchanged in comparison to control tissue [48].

It has previously been shown in rats that transcriptional activity of Nrf2 physiologically decreases with age [49]. One could therefore assume that the reduced Nrf2 mRNA and protein levels in ALS were age-dependent. As we used age-matched control tissues, however, loss in Nrf2 mRNA and protein expression cannot simply be explained by age effects. In contrast to studies in the ALS mouse model, we observed a decrease in Nrf2 expression in sporadic ALS postmortem tissue specimens and therefore suggested that this is associated with reduced cellular defense mechanisms against oxidative stress. A limitation of such postmortem studies is that they represent the terminal stage of ALS. The Nrf2 reduction which we observed does therefore not exclude activation of the Nrf2 pathway during disease onset or early symptomatic stages.

## 5. Preclinical Studies Using Nrf2 Activators

The Nrf2/ARE signaling system is a powerful defense system that evolved in higher organisms to protect them against an array of insults. The first assay to test the efficacy of Nrf2-dependent NQO1 activation in Hepa1c1c7 murine hepatoma cells was developed in 1988 [50]. Subsequently, 10 chemically distinct classes of Nrf2 activating compounds have been described, among them tert-butylhydroquinone, DL-sulforaphane, lipoic acid, fumaric acid, and curcumin [18]. Problems regarding these Nrf2-inducers include poor penetration of the blood-brain-barrier as well as their multifactorial modes of action not only related to the Nrf2-signaling cascade. Several studies have assessed the effects of different Nrf2-inducing agents in both wild-type and Nrf2-knockout mice and showed that different inducers result in differential gene expression changes and that not all gene expression changes are Nrf2-dependent (reviewed in [51]). 

Activation of Nrf2 is neuroprotective in animal models of ALS and other neurodegenerative diseases [29–39], (Table 1). One may predict that manipulation of Nrf2/ARE signaling is a potential novel mechanism for neuroprotection in humans against diseases such as ALS. In a preclinical study in transgenic ALS mice, we assessed a novel class of synthetic triterpenoids, that is, analogues of oleanolic acid which can be considered the most potent Nrf2-inducer to date [22]. We used the G93A SOD1 transgenic mouse model of ALS that is the best model available for ALS to date as it is well characterized and widely used to test therapeutic compounds against ALS. These mice develop symptoms of ALS and die from pathologies caused by SOD1 mutation at 85–90 days and 128–132 days after birth, respectively [5]. The synthetic triterpenoids we used in this study are analogs of 2-cyano-3, 12-dioxooleana-1,9-dien-28-oic acid (CDDO) derived from oleanolic acid. We used two CDDO-analogs, CDDO-EA and CDDO-TFEA, with an astounding potency of 2 × 10^5^ fold higher in activating Nrf2 and inducing phase II genes than the parent compound oleanolic acid. These compounds act at nanomolar levels in vitro and in vivo [29, 35, 52]. We have tested both of these CDDOs in G93A SOD1 mice and observed a significant increase in survival in mice treated with these compounds [29]. We noticed lower Nrf2 expression in the untreated G93A spinal cord sections as compared to the triterpenoid-treated group. This suggests that physiological activation of Nrf2 by oxidative stress does not take place in ALS transgenic mice. This could be due to multiple factors, such as the lack of or damage to other intermediary factors necessary for Nrf2 activation. Our study also used NSC-34 motor neuron-like cells stably expressing G93A SOD1. In this in vitro model, treatment with CDDO-EA or CDDO-TFEA led to increased Nrf2 protein expression as we showed by immunohistochemistry and Western blot. Triterpenoid-induced activation of Nrf2-expression could also be demonstrated in rat primary motor neurons. 

Total RNA analysis from CDDO and vehicle-treated NSC-34 G93A cells showed upregulation of classical Nrf2 regulated genes which could be divided into three categories: (1) antioxidants, (2) anti-inflammatory, and (3) genes related to mitochondrial protection. Both CDDOs increased NAD(P)H: quinone oxidoreductase (NQO1), glutathione S-transferase A3 (GSTa3), and heme oxygenase 1 (HO1) which are representatives of antioxidants. Cyclooxygenase-2 (Cox-2), inducible nitric oxide synthase (iNOS), Fas ligand (FasL), and tumor necrosis factor-alpha (TNF-alpha) expressions were similarly reduced by both CDDO analogs, showing that the treatment also affected expression of proinflammatory mediators. The third class of genes that we examined were factors involved in mitochondrial repair and biogenesis: here we found treatment-induced upregulation of cytochrome oxidase subunit II (COXII), estrogen-related receptor alpha (ERR-alpha), and peroxisome proliferator-activated receptor gamma coactivator 1-alpha (PGC-1alpha). Consistent to mRNA analysis, Western blot analysis showed increased expression in protein levels for Nrf2, NQO1, HO-1, and glutathione reductase (GR) in NSC-34 G93A SOD1 cells treated with CDDO-TFEA.

In the preclinical in vivo study we showed that CDDOs are bioavailable and penetrate the blood-brain barrier in mice. We also found no obvious toxicity during chronic administration of 80 mg/kg body weight. Treatment of G93A SOD1 mice treated with CDDO-EA and CDDO-TFEA begun at a presymptomatic age resulted in a significant increase in survival of 20.6 and 17.5 days, respectively. Treatment with these two compounds at the onset of ALS which is more relevant with respect to further translation into clinical studies in ALS patients still shows encouraging results: starting treatment at symptomatic age significantly extended the duration from age of onset to age of death by 43% (CDDO-EA) and 38% (CDDO-TFEA) [29]. 

**Figure 2:**
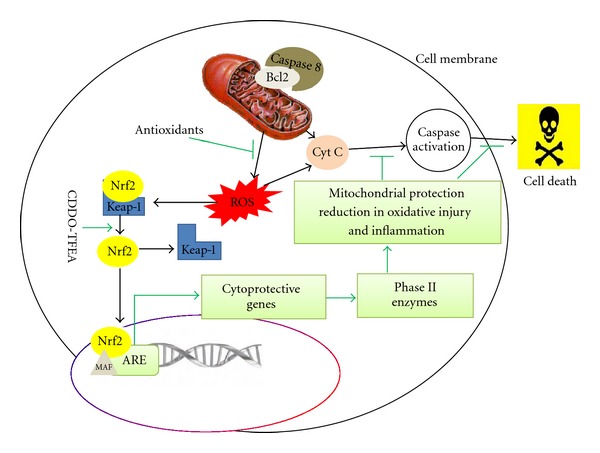
Schematic representation of major pathways involved in motor neuron death. The Nrf2/ARE signaling pathway is a potential target for blocking multiple death pathways. Green arrows represent neuroprotective pathways, and black arrows point to known neuronal death pathways containing potential cellular targets for antioxidant and anti-inflammatory agents.

In both paradigms, treatments attenuated weight loss and preserved motor performance. These Nrf2-activating compounds therefore represent interesting candidates for further clinical evaluation in ALS. Thus far, any investigated approaches for treating neurodegenerative diseases have targeted only one pathway or a specific cell type (e.g., astrocytes) to be blocked, whereas it is known that multiple and cascading pathways act in a noncell autonomous manner that leads to motor neuron death in ALS. To combat pathogenesis with this complexity and magnitude, we must aim at targets that are potentially powerful enough to block multiple pathways. Nrf2 induces over 250 phase II genes, and we have proof-of-concept that multiple genes are active following treatment with CDDO-EA or TFEA, some that produce antioxidant enzymes against oxidative stress, some that produce anti-inflammatory enzymes that target inflammation, and some that produce mitochondrial protective and repair enzymes that target dysfunctional mitochondria (Figure 2). Interestingly, another laboratory consistently demonstrated the neuroprotective effect of Nrf2 in MPTP model of Parkinson's disease [53].

## 6. Perspectives

Simultaneous blockage of disease-specific broad toxic signaling cascades in motor neurons and glia may ultimately lead to more efficient neuroprotection in ALS. Stimulation of defense mechanisms that modulate neuroprotective genes which affect both neuronal and glial functions is a novel therapeutic approach and holds great promise. A key molecule to affect a variety of defense mechanisms is the transcription factor Nrf2 which activates the Nrf2/ARE signaling program. Nrf2 acts as master regulator of the cellular antioxidant response by stimulation of over 250 phase II genes that should be referred to as “prolife genes” since they save cells from death. Nrf2 activation can at once regulate the expression of multiple cytoprotective enzymes that are capable of simultaneous inhibition of major pathogenic pathways described in ALS such as oxidative stress, neuroinflammation, and mitochondrial dysfunction. Decreased Nrf2 expression was found in motor neurons in ALS postmortem brain and spinal cord. We have established the proof-of-concept that the Nrf2/ARE program is a viable target with excellent therapeutic potential for ALS and have demonstrated that activation of Nrf2 by CDDOs resulted in significant beneficial effects on body weight, motor performance, and survival in the G93A SOD1 mouse model of ALS. While there are still multiple gaps of knowledge on the path from Nrf2 dissociation to nuclear localization and its action as transcription factor, activation of the Nrf2 signaling cascade represents a novel and unique attempt to find a cure for ALS and other neurodegenerative diseases by fortifying the intrinsic defense mechanisms of neurons. 

## Figures and Tables

**Table 1:** Evidence for protective effects of Nrf2-activation in animal models of neurodegenerative disorders.

Disease	Animal model	Method of Nrf2-activation	Reference
ALS	G93A-SOD1 mice	Synthetic triterpenoids (CDDO-ethylamide (CDDO-EA), CCDO-trifluoroethylamide (CDDO-TFEA))	Neymotin et al., 2011 [29]
Alzheimer's disease (AD)	Intracerebroventricular infusion of Abeta peptides in rats	Curcumin	Frautschy et al., 2001 [30]
AD	Transgenic APP/PS1 mice	Tert-butylhydroquinone/adenoviral Nrf2 gene transfer	Kanninen et al., 2008 [31]
AD	Transgenic 19959 mice	CDDO-methylamide (CDDO-MA)	Dumont et al., 2009 [32]
AD	Transgenic APP/PS1 mice	Intrahippocampal injection of Nrf2-expressing lentiviral vector	Kanninen et al., 2009 [33]
Parkinson's disease (PD)	MPTP-toxicity (mice)	Tert-butylhydroquinone	Abdel-Wahab, 2005 [34]
PD	MPTP-toxicity (mice) 3-NP-toxicity (rats)	CDDO-MA	Yang et al., 2009 [35]
PD	MPTP-toxicity (mice)	Astrocytic Nrf2-overexpression	Chen et al., 2009 [36]
PD	Alpha-synuclein expressing *Drosophila *	Nrf2-overexpression, Keap1-downregulation	Barone et al., 2011 [37]
Huntington's disease (HD)	Transgenic N171-82Q mice	CDDO-EA, CDDO-TFEA	Stack et al., 2010 [38]
HD	CAG 140 knock in mice	Curcumin	Hickey et al., 2012 [39]
